# Atlanta Medical College

**Published:** 1860-03

**Authors:** 


### Atlanta Medical College.

The next Session of the Atlanta Medical College commences on the first Monday in May, and is approaching with unprecedented prospects for a large class. The anatomical rooms will be opened by the 15th of April.

